# PERSONALIZED DEMENTIA PREVENTION IN LUXEMBOURG: INSIGHTS INTO THE PROGRAMME DEMENTIA PREVENTION (PDP)

**DOI:** 10.1093/geroni/igad104.3520

**Published:** 2023-12-21

**Authors:** Valerie Schröder, Amna Skrozic, Dorothee Erz, Anne Kaysen, Joëlle Fritz, Joao Loureiro, Deborah McIntyre, Rejko Krueger

**Affiliations:** University of Luxembourg, Esch-sur-Alzette, Grevenmacher, Luxembourg; University of Luxembourg, Esch-sur-Alzette, Grevenmacher, Luxembourg; University of Luxembourg, Esch-sur-Alzette, Grevenmacher, Luxembourg; University of Luxembourg, Esch-sur-Alzette, Grevenmacher, Luxembourg; Luxembourg Institute of Health, Luxembourg, Grevenmacher, Luxembourg; Centre Hospitalier de Luxembourg (CHL), Luxembourg, Grevenmacher, Luxembourg; Luxembourg Institute of Health, Luxembourg, Grevenmacher, Luxembourg; University of Luxembourg, Esch-sur-Alzette, Grevenmacher, Luxembourg

## Abstract

**BACKGROUND:**

Given that in an increasingly ageing society, the number of people with cognitive impairment is expected to increase, prevention strategies to reduce the high personal and societal burden of neurodegenerative diseases leading to dementia is of essence.

**METHODS:**

As part of the “Dementia Prevention Programme (pdp)” in Luxembourg, individuals with Mild Cognitive Impairment (MCI) or Subjective Cognitive Decline (SCD) are recruited via referring physicians and other sources. All participants undergo a comprehensive neuropsychological evaluation and a risk factor profile is created. Factors known to increase the risk to develop dementia include hypertension, hypercholesterolaemia, physical or cognitive inactivity, depression, social isolation or hearing loss. Based on the results of this analysis, personalised lifestyle interventions are recommended to each participant, such as cognitive training, physical activities, psychological counselling or nutritional advice.

**RESULTS:**

To date, 491 participants (mean age = 69.0 years; SD = 11.2) have been recruited at various sites across the country, of whom 424 (86.4%) present with MCI and 42 (8.6%) with SCD. 25 participants (5.1%) showed more advanced cognitive difficulties associated with loss of autonomy or had neither subjective nor objective cognitive difficulties and thus did not belong to the target group.

**CONCLUSION:**

We provide evidence for the successful implementation of a nationwide dementia prevention programme and efficient recruitment of the target population by building a network of different health care providers. This may serve other countries as an example to rule out similar programmes targeting at promoting healthy ageing among the general population.

